# MOGAD-Associated Recurrent Optic Neuritis Unresponsive to Corticosteroids: Clinical, Visual Field, and OCT Correlation

**DOI:** 10.22336/rjo.2026.21

**Published:** 2026

**Authors:** Cansu Kostakoğlu Duman, Büşra Gülhan, Yelda Yıldız Taşçı, Gönül Vural

**Affiliations:** 1 Department of Neurology, Ankara Bilkent City Hospital, University of Health Sciences, Ankara, Turkey; 2 Department of Ophthalmology, Ankara Bilkent City Hospital, University of Health Sciences, Ankara, Turkey; 3 Department of Neurology, Ankara Bilkent City Hospital, Ankara Yıldırım Beyazıt University, Ankara, Turkey

**Keywords:** optic neuritis, myelin oligodendrocyte glycoprotein, demyelinating disease, differential diagnosis, BCVA = Best-Corrected Visual Acuity, CRION = Chronic Relapsing Inflammatory Optic Neuropathy, CSF = Cerebrospinal Fluid, IV = Intravenous, IVIG = Intravenous Immunoglobulin, MRI = Magnetic Resonance Imaging, MOG = Myelin Oligodendrocyte Glycoprotein, MOGAD = Myelin Oligodendrocyte Glycoprotein Antibody-Associated Disease, MS = Multiple Sclerosis, NMOSD = Neuromyelitis Optica Spectrum Disorder, OCB = Oligoclonal Bands, OCT = Optical Coherence Tomography, OD = Optic Disc, ON = Optic Neuritis, RNFL = Retinal Nerve Fiber Layer, VEP = Visual Evoked Potential

## Abstract

**Background:**

Myelin oligodendrocyte glycoprotein antibody-associated disease (MOGAD) is a rare autoimmune demyelinating disorder. In adults, optic neuritis is the most common initial manifestation.

**Case presentation:**

We report the case of a 23-year-old woman presenting with right eye vision loss and severe optic disc edema. The condition was unresponsive to intravenous corticosteroids. Plasmapheresis led to marked improvement. Two months later, a second episode occurred in the left eye, despite ongoing steroid therapy.

**Investigations:**

Initial brain and orbital MRI were normal. Optical coherence tomography (OCT) showed increased RNFL thickness in the acute phase and progressive thinning during follow-up. Anti-MOG antibodies were detected at a 1:100 titer in serum, confirming the diagnosis.

**Treatment and outcome:**

The patient was treated with plasmapheresis, oral steroids, and azathioprine during the first attack. After the second relapse, rituximab was initiated. The patient achieved visual recovery and has remained relapse-free for two years.

**Conclusion:**

Corticosteroid-unresponsive recurrent optic neuritis should prompt evaluation for MOGAD, especially in the presence of bilateral involvement and optic disc edema. OCT is valuable for monitoring disease activity and structural damage.

## Introduction

Optic neuritis (ON) is an inflammatory condition of the optic nerve characterized by painful unilateral vision loss and may be associated with systemic or neurological disorders such as Multiple Sclerosis (MS), Neuromyelitis Optica Spectrum Disorder (NMOSD), and Myelin Oligodendrocyte Glycoprotein Antibody-Associated Disease (MOGAD); in cases of recurrence, MOGAD, NMOSD, and Chronic Relapsing Inflammatory Optic Neuropathy (CRION) should be considered in the differential diagnosis [1]. MOGAD is a demyelinating disease characterized by the formation of antibodies against myelin oligodendrocyte glycoprotein (MOG), a membrane protein expressed on the surface of oligodendrocytes. Updated diagnostic criteria were published in 2023, and the key diagnostic criterion is a positive MOG antibody in serum, as detected by cell-based assays [2]. The estimated annual incidence of MOGAD is approximately 1.6-4.8 cases per million, with a global prevalence of 1.3-2.5 cases per 100,000 population [3]. There is a biphasic distribution peaking in childhood and early adulthood [2,3]. Optic neuritis, observed in 30–60% of adult patients, is the most common clinical manifestation of MOGAD and typically presents with bilateral involvement, optic disc swelling, and optic perineuritis features that help differentiate it from other demyelinating disorders [3]. Magnetic resonance imaging for the diagnosis of central nervous system lesions and optical coherence tomography for the demonstration of retinal structural damage and degeneration are also significant in the differential diagnosis [4]. Intravenous corticosteroids are the first-line treatment for acute MOGAD-ON attacks; however, corticosteroids have been reported to accelerate visual recovery but not to alter long-term visual outcomes [5]. More aggressive therapies, such as plasmapheresis and IVIG, can be tried if adequate visual improvement is not achieved with corticosteroids [1,5]. Oral corticosteroids, azathioprine, mycophenolate mofetil, and rituximab can be used in maintenance treatment [5]. We present a case of MOGAD with corticosteroid-unresponsive recurrent optic neuritis, supported by color fundus imaging, visual field testing, and optical coherence tomography findings.

## Case report

**Fig. 1:**
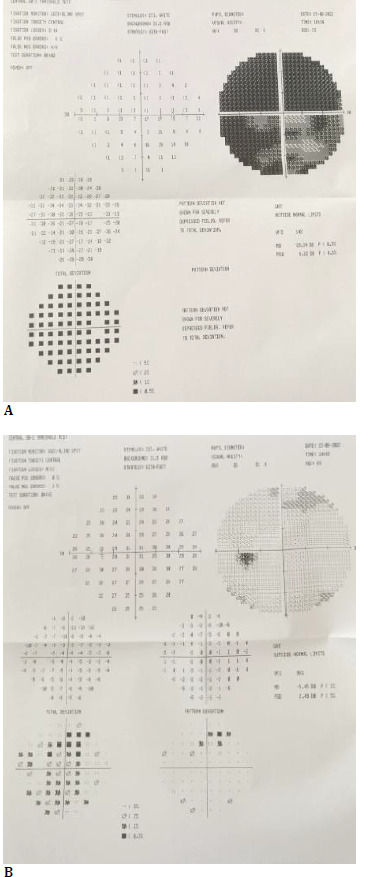
30-2 visual field test at the patient’s first application (**A**: Right eye, **B**: Left eye)

A 23-year-old female patient with no known history of comorbidities applied to the clinic with blurred vision in the right eye lasting 2 weeks and intermittent, throbbing headache in the frontal region and spreading to the periorbital regions. Neurological examination revealed anisocoric pupils and relative afferent pupillary defect in the right eye. On ophthalmologic examination, best-corrected visual acuity (BCVA) on the Snellen chart was 0.4 in the right eye and 1.0 in the left eye. Relative afferent pupillary defect was positive in the right eye. Color vision was 0/12 in the right eye and 12/12 in the left eye with Ishihara charts. Biomicroscopy showed a mid-dilated anterior segment, right pupil, and anisocoria. Fundus examination revealed an edematous right optic disc (OD) and blurred nasal left OD borders. In the 30-2 visual field test, a near-total absolute visual field defect was observed on the right, and a superior visual field defect was observed on the left due to the upper eyelid effect (**Fig. 1A, B**).

**Fig. 2:**
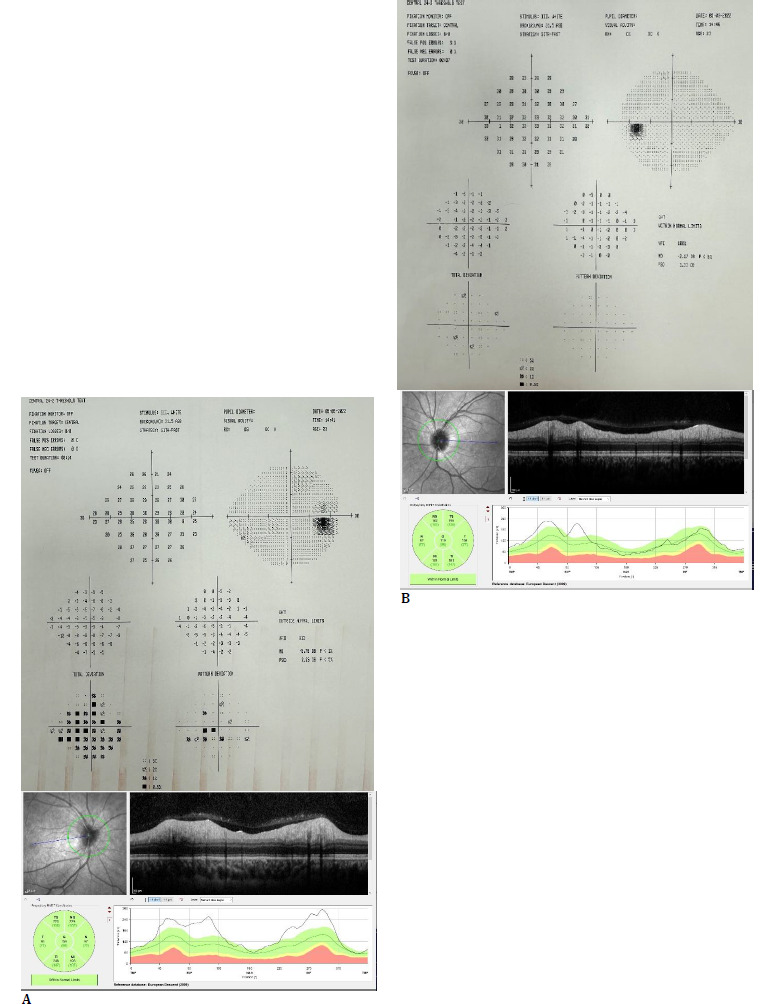
The patient’s 24-2 visual field test and retinal nerve fiber analysis with optical coherence tomography two weeks after the first attack (**A**: Right eye, **B**: Left eye)

Routine laboratory tests were normal. No pathology was observed in the brain, orbital, cervical, or thoracic magnetic resonance imaging. The visual evoked potential (VEP) test was within normal limits on the left, while conduction delay and amplitude loss were detected in the right anterior visual pathways. Laboratory panel for vasculitis exclusion was negative. Cerebrospinal fluid (CSF) examinations revealed normal opening pressure, cell count, and total protein. The patient received 1 gr/day IV methylprednisolone treatment for 10 days. The patient did not describe visual improvement. After corticosteroid treatment, visual acuity, biomicroscopy, and fundus examination were similar. 24-2 Humprey visual field test (Carl Zeiss Meditec Inc., Dublin, CA) revealed widening of the blind spot and central visual field defect in the right eye, normal limits in the left eye; optical coherence tomography (OCT, Heidelberg, Germany) revealed total retinal nerve fiber thickness of 159 and 119 µm in the right and left eyes, respectively (**Fig. 2A, B**).

**Fig. 3:**
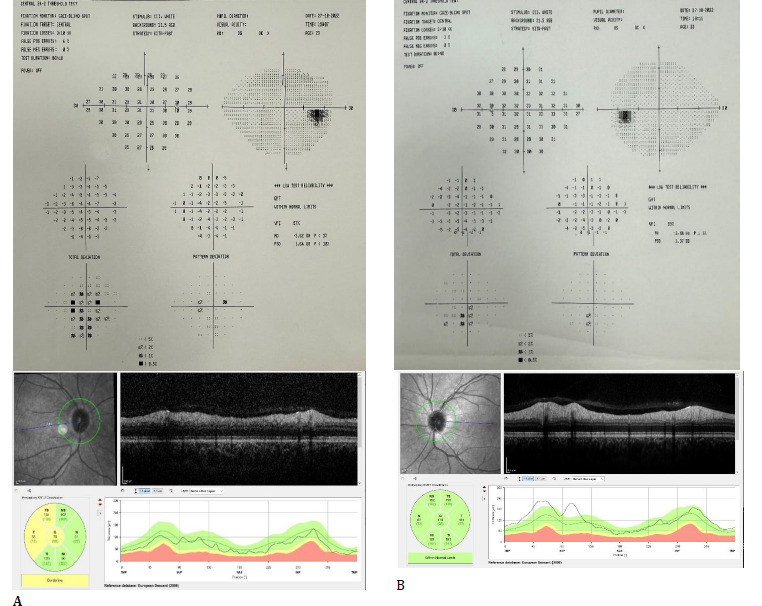
The patient’s 24-2 visual field test and retinal nerve fiber analysis with optical coherence tomography six weeks after the first attack (**A**: Right eye, **B**: Left eye)

Anti-aquaporin 4 antibody (NMO IgG) was negative in serum and CSF, CSF Oligoclonal band (OCB) was negative, and anti-MOG protein was positive at a 1/100 titer, and MOGAD-ON was diagnosed. After corticosteroid treatment ended, the patient received plasmapheresis every other day for 5 days. After plasmapheresis, the patient's vision improved significantly, and she was discharged with maintenance oral steroids and azathioprine 100 mg/day. At the 6-week follow-up, BCVA was 1.0 bilaterally, color vision was 12/12 bilaterally, and relative afferent pupillary defect was positive in the right eye. Biomicroscopic anterior segment was bilaterally normal, fundus examination showed the right OD was slightly pale, and the left OD borders were slightly blurred nasally. In the 24-2 visual field test, the blind spot in the right eye was widened, and the left eye was within normal limits, and the mean RNFL was 78 and 101 µm in the right and left eyes, respectively (**Fig. 3A, B**).

**Fig. 4:**
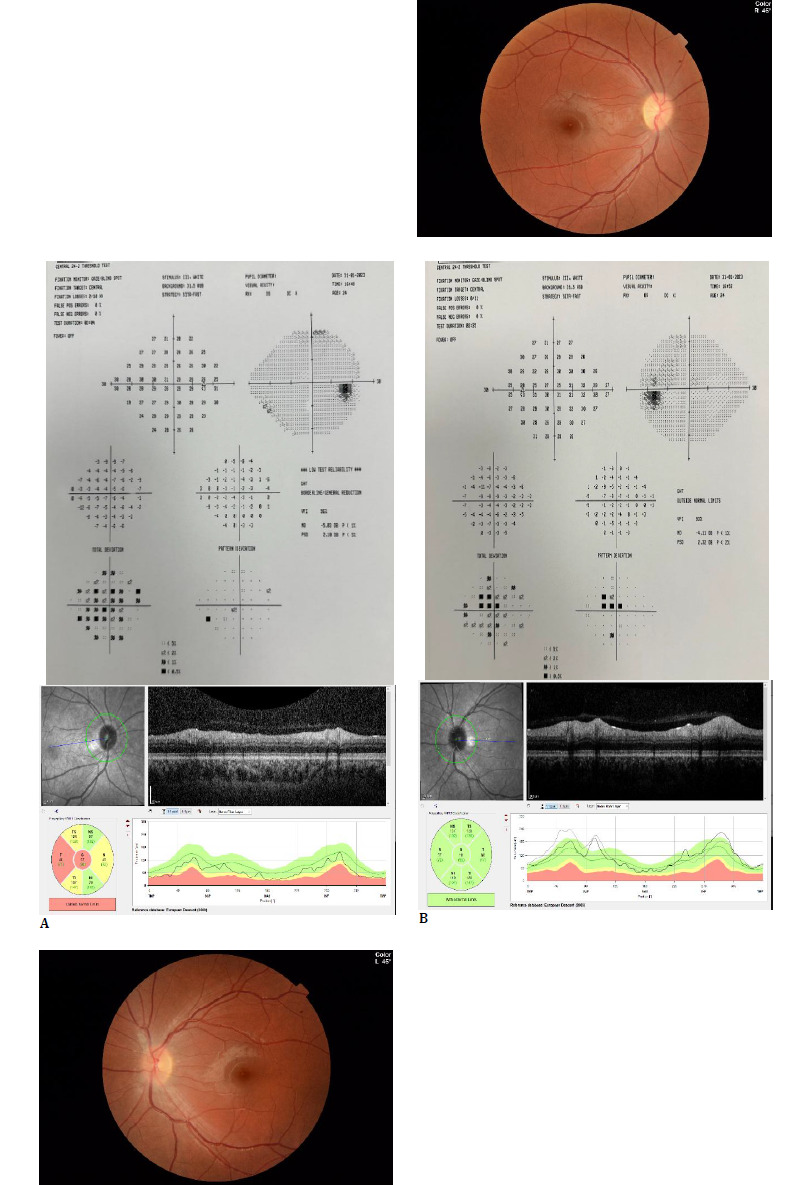
The patient’s Color fundus photograph, 24-2 visual field test, and retinal nerve fiber analysis with optical coherence tomography in the second attack (**A**: Right eye, **B**: Left eye)

While on maintenance oral corticosteroid therapy, the patient presented with a new attack of blurred vision in the left eye 2 months later. BCVA in the right eye was 1.0, left eye was 0.7, color vision in the right eye was 12/12, left eye was 10/12, and relative afferent pupillary defect was positive in the right eye. Biomicroscopic anterior segment was bilaterally normal. Fundus examination showed right OD was pale, left OD borders were blurred in the nasal, superior, and inferior directions; visual field was within normal limits on the right, there was blind spot enlargement in the left eye, and a paracentral visual field defect. Mean RNFL was 67 and 111 µm in the right and left eyes, respectively (**Fig. 4A, B**).

**Fig. 5:**
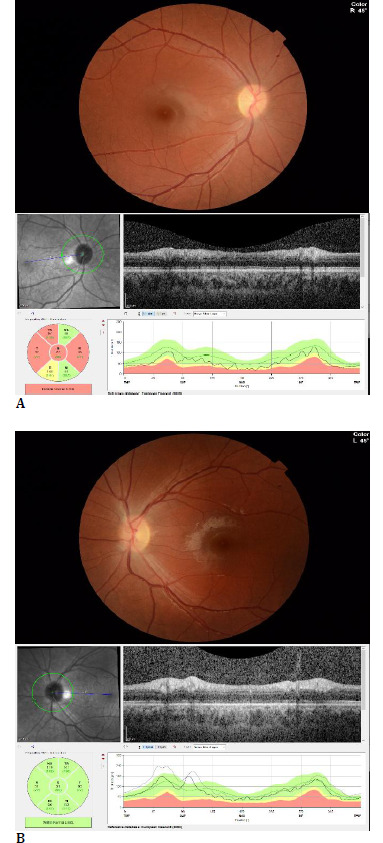
The patient’s Color fundus photograph and retinal nerve fiber analysis with optical coherence tomography at the last follow-up (**A**: Right eye, **B**: Left eye)

There were no additional findings in the neurological examination. Given the lack of response to corticosteroids during the first episode, plasmapheresis was performed every other day for 5 days again, and rituximab was planned. The patient's vision improved, and at the ophthalmological examination 4 weeks after the second attack, bilateral BCVA was 1.0; color vision was 12/12 in the right eye and 10/12 in the left eye; and a relative afferent pupillary defect was present in the right eye. Biomicroscopic anterior segment was bilaterally normal, fundus examination showed right OD was pale, and left OD borders were blurred nasally. Mean RNFL was 67 and 91 µm in the right and left eyes, respectively (**Fig. 5A, B**).

The patient received 1000+1000 mg rituximab at 15-day intervals, and 1000 mg rituximab was repeated once every 6 months after the first course. Verbal and written consent were obtained from the patient for data sharing. The patient has been followed up without attacks for the last two years.

## Discussion

Eye pain of MOGAD-ON may radiate from the ocular area to the periorbital area and then to the frontotemporal regions, as in our case. Considering that MOGAD is often bilateral, if it presents with optic disc edema at first presentation and is accompanied by headache, it can be interpreted in favor of papillary stasis and suggest increased intracranial pressure [3]. Also, brain magnetic resonance imaging can yield normal results in 50% of adult MOGAD patients; in this case, the key point [6] is that, from a neurological perspective, these patients present with acute vision loss. In contrast, in cases of elevated intracranial pressure (such as idiopathic intracranial hypertension), vision loss typically occurs in the advanced stages. It presents as concentric visual field constriction with preserved central vision [7]. It should be noted that normal brain and orbital magnetic resonance imaging (MRI) does not exclude demyelinating disease [1]. Visual evoked potentials may demonstrate a delayed P100 latency and decreased amplitude [1,2]. Retinal structural damage and degeneration in MOGAD-ON can be measured with optical coherence tomography (OCT), a noninvasive technique that quantifies neuroaxonal loss by assessing changes in intraretinal layer thickness or volume [4]. Thus, OCT may serve as both a confirmation of optic neuritis and a means of differentiating MOGAD from other demyelinating diseases, such as MS [8,9]. According to Chen et al., the median pRNFL was 164 μm in 96 acute MOGAD ON eyes, while it was 103 μm in 51 acute MS ON eyes [8]. According to Sechi et al., in MOGAD_ON, after 3-6 months, there is progressive thinning of the pRNFL, macular ganglion cell, and inner plexiform layer (mGCIPL); thinning in mGCIPL occurs earlier and within a few weeks after the attack, while thinning in pRNFL lasts longer, probably due to gradually resolving optic nerve head swelling [9]. It has been reported in the literature that MOGAD-ON attack responds well to high-dose intravenous corticosteroids, but in our case, no adequate clinical response was observed [3]. According to a multicenter study on plasmapheresis for optic neuritis published in 2023, plasmapheresis in MOGAD-ON was associated with a favorable prognosis [10]. Initiating which immunosuppressive treatment after the first attack in MOGAD-ON is unclear; we generally treat acute attacks with high-dose corticosteroids in our clinical practice. If the response is insufficient, we proceed with plasmapheresis, as 71.2% of clinicians in a recent international survey [11] did. However, due to the relapsing nature of the disease in our patient, we initiated aggressive treatment with plasmapheresis immediately during the second attack. Considering that this was a new episode of optic neuritis, azathioprine may not have reached therapeutic efficacy yet, and maintenance corticosteroids alone were insufficient to control the attack. Subsequently, rituximab was initiated as part of a more aggressive treatment strategy. The patient was followed up under rituximab treatment without any attacks. It remained unclear whether that outcome reflected the natural course of the disease or the effect of our therapeutic approach.

## Conclusion

MOGAD-associated optic neuritis is rare. Its follow-up and treatment require attention. It should definitely be kept in mind in patients presenting with acute vision loss and optic disc edema, and its characteristic features, especially bilateral involvement and tendency to relapse, should be known.

## Data Availability

**The data supporting the findings of this study are available from the corresponding author upon reasonable request**.
